# 3D imaging of PSD-95 in the mouse brain using the advanced CUBIC method

**DOI:** 10.1186/s13041-018-0393-4

**Published:** 2018-09-12

**Authors:** Huazheng Liang, Hongqin Wang, Shaoshi Wang, Richard Francis, George Paxinos, Xufeng Huang

**Affiliations:** 1 Neurology Department, Shanghai No.1 People’s Hospital (in the process of affiliation), School of Medicine, Tongji University, Shanghai, 200081 China; 2 Neuroscience Research Australia, Randwick, NSW 2031 Australia; 3 School of Medicine, Western Sydney University, Campbelltown, NSW 2560 Australia; 4 Illawarra Health and Medical Research Institute, Wollongong, NSW 2522 Australia; 5 Biomedical Imaging Facility, Mark Wainwright Analytical Centre, The University of New South Wales, Kensington, NSW 2052 Australia

**Keywords:** PSD-95, Mouse, Advanced CUBIC, Transparent brain, Immunofluorescent staining

## Abstract

**Aims:**

Postsynaptic density – 95 kDa protein (PSD95) is an important molecule on the postsynaptic membrane. It interacts with many other proteins and plays a pivotal role in learning and memory formation. Its distribution in the brain has been studied previously using in situ *hybridization* as well as immunohistochemistry*.* However, these studies are based on 2 dimensional (2D) sections and results are presented with a few sections. The present study aims to show PSD-95 distribution in 3 dimensions (3D) without slicing the brain tissue of C57BL/6 mice into sections using the advanced CUBIC technique.

**Methods:**

Immunofluorescent staining using a PSD-95 antibody was performed on a half of the mouse brain after clarifying it using the advanced CUBIC protocol. The brain tissue was imaged using a Zeiss Z1 light sheet microscope and 3D reconstruction was completed using the Arivis Vision 4 dimensional (4D) software.

**Results:**

The majority of brain nuclei have similar distribution pattern to what has been reported from in situ *hybridization* and immunohistochemical studies in the mouse. The signal can be easily followed in the 3D and their spatial relationship with adjacent structures clearly demarcated. In the present study, some fiber bundles also showed strong PSD-95 signal, which is different from what was shown in previous studies and need to be confirmed in future studies.

**Electronic supplementary material:**

The online version of this article (10.1186/s13041-018-0393-4) contains supplementary material, which is available to authorized users.

## Main text

Postsynaptic density protein of 95 kDa (PSD-95) is a scaffolding protein encoded by the disc large homolog 4 (DLG4) gene. It is highly enriched in the postsynaptic membrane and interacts with multiple synaptic proteins [1] through its three Post synaptic density protein, Drosophila disc large tumor suppressor, and Zonula occludens-1 protein (PDZ) domains. It plays an important role in synaptic plasticity [2], learning and memory [3], and other functions depending on the proteins it binds to. The expression of PSD-95 in the mouse brain has been described in traditional studies [4–6]. PSD-95 is highly expressed in the cerebral cortex, hippocampus, and striatum, and only moderately or weakly expressed in other brain regions. Our research has focused on the molecular and histological changes in the brain of disrupted in schizophrenia 1-locus impairment (DISC1-LI) mice, which is known to be relevant to the neuropathology of schizophrenia. The 3D imaging results presented here were from the C57BL/6 mouse as part of our histological analysis comparing the expression changes of PSD-95 in DISC1-LI mice with that of control C57BL/6 mice in the 3D space.

Brains of 12-week old C57BL/6 male mice were fixed with 4% paraformaldehyde and dissected before being cut into two halves sagittally. Immunofluorescent staining using a PSD-95 antibody was performed on a half of the brains after clarifying them using the advanced CUBIC protocol [7]. The brain tissue was imaged with a Zeiss Z1 light sheet microscope using a 20× clearing objective and 3D reconstruction was completed using the Arivis Vision 4 dimensional (4D) software.

**Fig. 1:**
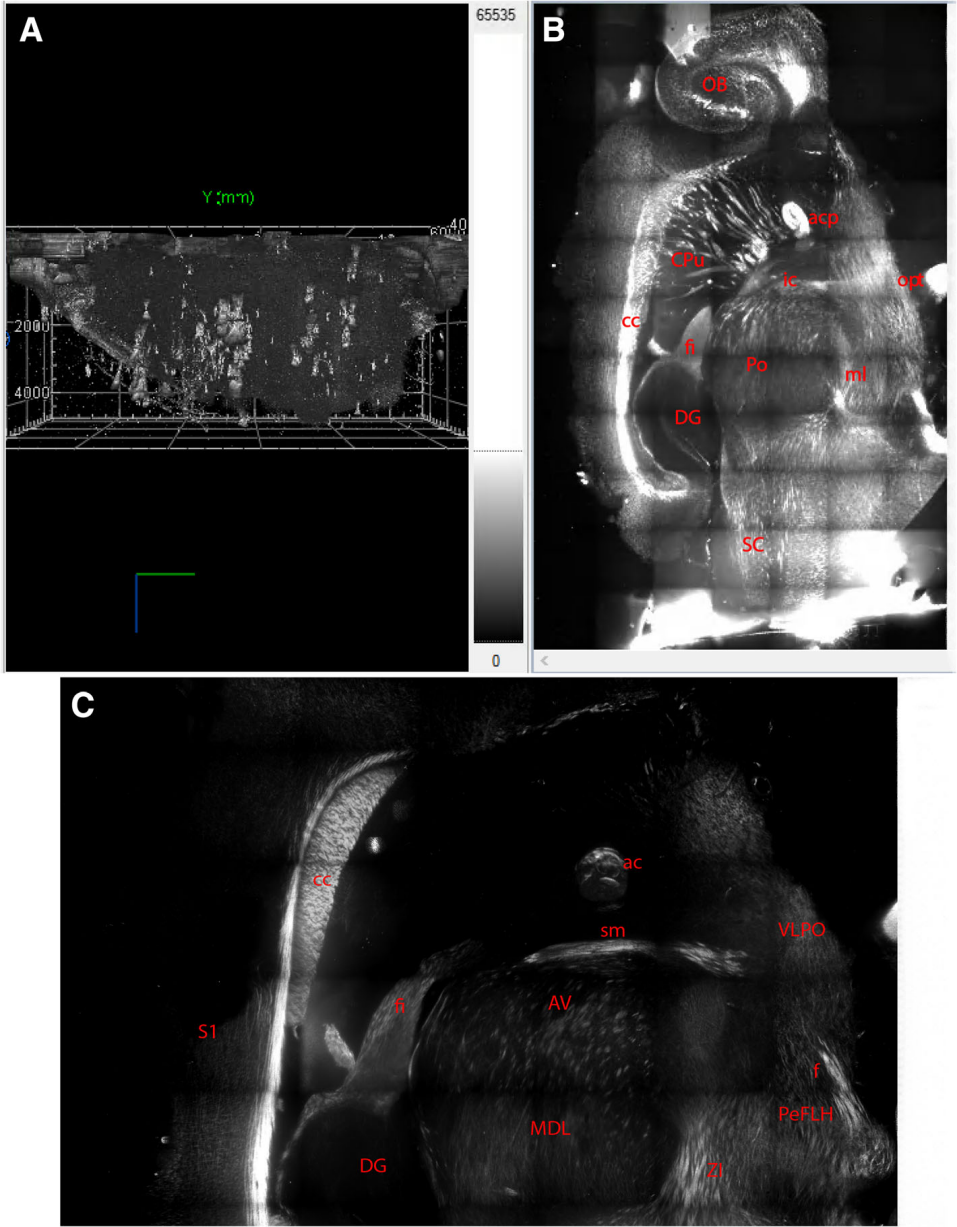
Fluorescent staining with a PSD-95 antibody on the clarified mouse brain. **a**. PSD-95 positive signal in a 3D rendered image. **b**. PSD-95 positive signal in a 2D image. The top is the rostral part, the bottom is the caudal part, the left is the dorsal part, and the right is the ventral part of the mouse brain. **c**. PSD-95 positive signal in the forebrain. Note: strongly positive signal in the corpus callosum, anterior commissure, fornix; moderate signal in other brain areas. The gray matter of the hippocampus does not have PSD-95 positive signal

In the forebrain, we found strong positive PSD-95 signal in the internal plexiform and the mitral cell layers of the olfactory bulb (Fig. 1a and b), which was similar to what has been reported in the in situ *hybridization* [4, 8, 9] and immunohistochemistry studies [5, 6].

Ventral to the cerebral cortex, which had weak PSD-95 signal, moderate signal was observed in the corpus callosum except the area dorsal to the hippocampus (Fig. 1b and c). The positive signal in this stripe was aligned in the same direction as that observed in the caudate putamen, radiating towards the meeting point of the stria terminalis and the internal capsule, which is consistent with results from other studies [4–6, 9]. The hippocampal fissure showed a narrow line of weak signal (Fig. 1b and c), which is in contrast to what was reported in a study using lacZ [6]. In a human postmortem study, it was reported that schizophrenia and bipolar patients showed a lower level of PSD-95 in the hippocampus, especially in the molecular layer of the dentate gyrus, suggesting the diagnostic value of PSD-95 in the reported diseases [10].

Strong signal was also found in the anterior part of the anterior commissure, the fornix, the optic nerve, and the cerebral peduncle ventral to the substantia nigra (Fig. 1b and c). This is in a clear contrast to what has been reported in the lacZ gene knockin mice [6] and other in situ hybridization [4, 9] as well as immunohistochemical studies [5]. In those studies, most if not all fiber tracts are void of PSD-95 signal. Moderate signal was observed in the shell of accumbens nucleus, the ventral pallidum, and the nucleus of horizontal diagonal band, the lateral preoptic area, and the majority of hypothalamic nuclei. In the thalamus, its rostral half had moderate PSD-95 signal, whereas the caudal half did not have or had weak PSD-95 signal (Fig. 1b and c). This is different from what has been reported by Porter et al. [6].

Positive signal was observed in the majority of midbrain nuclei and nuclei in the rostral hindbrain (the rest of the hindbrain and cerebellum were cut off). Positive signal in the midbrain and the hypothalamus tended to follow the course of large fibers travelling rostrocaudally. In the middle part of the midbrain in sagittal sections, a small band of signal appeared to travel dorsoventrally. Ventral to this band, positive signals converge towards the ventral surface of the hindbrain (Fig. 1b). These features were not observed in previous in situ *hybridization* and immunohistochemical studies.

The present study showed the expression of PSD-95 in the mouse brain using an emerging 3D technique – advanced CUBIC and this study confirmed some previous reports about PSD-95 in the majority of brain nuclei (Additional file 1). We showed that some fiber bundles were positive for PSD95, which has not been reported in previous studies. To verify this concern, we used Western blot (not shown) and confirmed the specificity of our PSD95 antibody. Our finding might be true due to the fact that lipid of the neuropil has been removed by the clearing solution and the antibody can easily bind to the epitope of PSD-95, whereas in conventional sections, there is no such a step and the interaction between PSD-95 and its antibody may be blocked to some extent, especially in a fiber bundle where the fibers are tightly bound to each other.


**Additional file 1:** 3D video of fluorescent PSD-95 signal reconstructed using Arivis. Strong fluorescent signal was observed in the large fiber bundles such as the anterior commissure, fornix, stria terminalis of the thalamus, and corpus callosum. Weak to moderate signal was observed in a large number of nuclei in the forebrain and midbrain. (AVI 34006 kb)


The present study did not include the entire hindbrain due to difficulty in mounting the clarified tissue, which was very soft, to the glass capillary for imaging. Based on the findings from the rostral hindbrain, which are similar to what has been shown in in situ *hybridization* studies [4, 9], it is expected that the majority of hindbrain nuclei will have weak to moderate PSD-95 signal. The cerebellum might have a higher level of signal than the other areas in the hindbrain as indicated by the in situ *hybridization* studies.

The advanced CUBIC is an efficient technique in clarifying the mouse brain tissue (Additional file 2). For the same reason, it might lead to more protein loss than passive Clear Lipid-exchanged Acrylamide-hybridized Rigid Imaging/ Immunostaining/In situ hybridization-compatible Tissue hYdrogel (CLARITY) and other similar techniques. Currently, no method is ideal for preserving protein and rendering tissue transparent for imaging. Better clearing and imaging techniques with longer working distance will be in demand in order to show tissue integrity in the 3D video.

## Additional files

Additional file 2: Materials and methods. (DOC 30 kb)

## Abbreviations

2D: 2 dimensional
3D: 3 dimensions
4D: 4 dimensional
ac: Anterior commissure
acp: Anterior commissure, posterior part
AV: Anteroventral thalamic nucleus
cc: Corpus callosum
CLARITY: Clear Lipid-exchanged Acrylamide-hybridized Rigid Imaging/ Immunostaining/In situ hybridization-compatible Tissue hYdrogel
CPu: Caudate putamen
CUBIC: Clear, Unobstructed Brain/Body Imaging Cocktails and Computational analysis
DG: Dentate gyrus
DISC1-LI: Disrupted in schizophrenia 1-locus impairment
DLG4: Disc large homolog 4 gene
f: Fornix
fi: Fimbria of hippocampus
ic: Internal capsule
MAGUK: Membrane associated guanylate kinase
MDL: Lateral part of the mediodorsal thalamic nucleus
NA: Numeral aperture
OB: Olfactory bulb
opt: Optic tract
PBS: Phosphate-buffered saline
PC1: Physical containment level 1
PDZ: Post synaptic density protein (PSD95), drosophila disc large tumor suppressor, and zonula occludens-1 protein
PeFLH: Perifornical lateral hypothalamus
PFA: Paraformaldehyde
Po: Posterior thalamic nuclear group
PSD95: Postsynaptic density – 95 kDa protein
S1: Primary sensory cortex
SC: Superior colliculus
sm: Stria medullaris of the thalamus
VLPO: Ventrolateral preoptic nucleus
ZI: Zona incerta
