# Supplementary material for: 3D imaging of PSD-95 in the mouse brain using the advanced CUBIC method
Source: Mol Brain. 2018 Sep 12;11:50. doi: 10.1186/s13041-018-0393-4 (PMC6134724; doi:10.1186/s13041-018-0393-4)
Supplement: Supplementary file 2 — Materials and methods. (DOC 30 kb) [file 13041_2018_393_MOESM2_ESM.doc]

**Materials and methods**

**Animals**

Experiments were performed on 12-week old C57BL/6 (n=3) male mice, weighing 20-30 g. They were purchased from the Animal Resource Centre in Western Australia and housed in the polypropylene cages (dimensions 405 mm x 205 mm x 185 mm, floor area 501cm^2^) with 2-4 mice in each cage in a PC1 animal facility. The animal experiment was approved by the Animal Care and Ethics Committee of The University of New South Wales (approval number: 14/94A) and conducted in compliance with [the revised Animals (Scientific Procedures) Act 1986](http://www.legislation.gov.uk/ukpga/1986/14/contents) in the UK.

The mouse brain was cut into halves along the midline sagittally and half of the brain was used for immunofluorescent staining using a PSD-95 antibody, and the other half served as a negative control by skipping the primary antibody.

**Tissue clearing**

Mice were anaesthetized with a lethal dose of pentobarbital solution (0.1 ml, 200 mg/ml) and perfused with 4% paraformaldehyde (PFA) in phosphate-buffered saline (PBS) in a fume hood that was in a different room from the animal house. The mouse brain was removed from the skull and washed with 1xPBS. The brain was cut into halves along the midline and the hindbrain and the cerebellum were removed before being transferred to the Clear, Unobstructed Brain/Body Imaging Cocktails and Computational analysis (CUBIC) 1 solution (3.85 g urea and 3.85 g N,N,N',N'-tetrakis (2-hydroxypropyl) ethylenediamine, 2.31 g polyethylene glycol mono-p-isooctylphenyl ether/Triton X-100 in 5.38 ml distilled water) (Susaki et al., 2015) in a 37^o^C oven. The solution was changed every 3 days until the mouse brain became transparent.

**Immunofluorescent staining**

After washing the brain tissue with 1xPBS every 6hr for 24hr, the tissue was incubated in the primary antibody solution (PSD-95, Cat No: ab120931, Abcam, produced in goat, 1:100) containing 0.2% sodium azide and 0.5% Triton-X100 for 2 weeks on a rotator at 37°C. Every 4 days, half the amount of PSD-95 was added to the antibody solution. The brain tissue was subsequently washed with PBS every 6 h for 24 h and incubated in the secondary antibody solution (Alexa Fluor® 647 donkey anti-goat IgG, Thermofisher, Cat No: A-21447, 1:50) for 2 weeks on a rotator at 37°C. The tissue was washed after the secondary incubation in 1xPBS every 6h for 24h before being incubated in the CUBIC 2 solution (Susaki et al., 2015).

**Refraction index matching**

The CUBIC 2 solution contains 50% (w/v) sucrose, 25% (w/v) urea, 10% (w/v) 2,2′,2′'-nitrilotriethanol, and 0.1% (v/v) Triton X-100. The mouse brain was incubated in this solution for 2-4 days until it became transparent again.

**Imaging**

The brain tissue was imaged with a Zeiss Z1 light sheet microscope (Oberkochen, Germany) using a 20x clearing objective (numeral aperture-NA=1). Samples were excited using a 561nm laser light sheet, fluorescence emission was collected using a 585nm long-pass filter.  Samples were optically sectioned by moving the sample through the light sheet during imaging to create a z-stack.  To generate each z-plane image, sample fluorescence was imaged by sequentially exciting the sample using the left and right orientated light sheets and imaging with a PCO Edge 5.5 camera.  Sequential images (i.e. left and right light sheet excited) were merged using Zen light sheet software (Zeiss) to create a single fluorescent image for each z-plane.  The brain tissue was scanned to give a final resolution of 0.57µm/pixel with z-step size of 1 µm.  3D reconstruction was completed using the Arivis Vision 4 dimensional (4D) software (Unterschleißheim, Germany) and screenshots were taken when the 3D image was zoomed in and out.
